# Effect of Imaging Distance and Chicken Body Size on Infrared Thermal Camera Accuracy in Body Temperature Measurement

**DOI:** 10.3390/vetsci12111062

**Published:** 2025-11-05

**Authors:** Jamlong Mitchaothai, Achara Lukkananukool, Patcharaporn Suwor, Suneeporn Suwanmaneepong

**Affiliations:** 1 Office of Administrative Interdisciplinary Program on Agricultural Technology, School of Agricultural Technology, King Mongkut’s Institute of Technology Ladkrabang (KMITL), Bangkok 10520, Thailand; jamlong.mi@kmitl.ac.th (J.M.); patcharaporn.su@kmitl.ac.th (P.S.); suneeporn.su@kmitl.ac.th (S.S.); 2 Department of Animal Production Technology and Fisheries, School of Agricultural Technology, King Mongkut’s Institute of Technology Ladkrabang (KMITL), Bangkok 10520, Thailand

**Keywords:** chicken, rectal temperature, thermal imaging, infrared thermography, body size, distance, precision livestock farming

## Abstract

**Simple Summary:**

Monitoring body temperature is vital to chicken health, but rectal measurement, though accurate, is invasive and stressful for the animal. Thermal cameras provide a non-contact option, yet their accuracy can be affected by bird size and camera distance. This study tested 90 chickens (small, medium, and large) by using both rectal thermometry and a thermal camera from distances of 50, 75, and 100 cm. The results showed that the measurements with the thermal imaging camera consistently underestimated the rectal temperatures, especially in small birds and at greater distances. In medium-sized birds, we also observed underestimation, though less severe, while for large birds, we obtained the most reliable results, with readings closer to rectal values and stronger correlations, particularly at 50–75 cm. In summary, thermal cameras cannot fully replace rectal thermometers for precise measurement in individual chickens. However, they offer a quick, stress-free method for flock-level screening, especially in larger birds. As part of precision livestock farming, thermal imaging could help future farms improve poultry health and welfare while reducing handling stress.

**Abstract:**

The accurate monitoring of body temperature is critical to poultry health and welfare. Rectal thermometry, the conventionally employed method, is invasive and stressful. Infrared thermography (IRT) offers a non-invasive alternative, but its accuracy may be influenced by body size and camera-to-object distance. This study evaluated the efficiency of thermal imaging compared with rectal thermometry in measuring chicken body temperature, with a focus on the effects of body size and measurement distance. A cross-sectional, repeated-measures design was applied to ninety clinically healthy Buff Sussex chickens (*n* = 30 per size group: small, medium, and large). Each bird was imaged from three distances (50, 75, and 100 cm) by using a thermal camera (FLIR C5^®^), with rectal temperature (Omron MC-246^®^) serving as the reference, so that a total of 270 paired observations were analyzed. Agreement was assessed using Bland–Altman bias and limits of agreement (LOAs), root mean square error (RMSE), mean absolute error (MAE), Pearson correlation (*r*), and Lin’s concordance correlation coefficient (CCC). The results showed that rectal temperatures were consistent with the normal physiological range reported for healthy chickens (40.43–41.95 °C) and that thermal imaging showed systematic underestimation, particularly in small birds (bias of up to −2.65 °C and RMSE of 2.72 °C at 100 cm) and medium-sized birds (bias of −0.73 to −1.39 °C), with weak concordance (CCC ≤ 0.16). Measurements in large birds demonstrated the smallest bias (−0.76 to +0.16 °C), lower errors (MAE of 0.73–0.89 °C), and stronger correlations (*r* = 0.56–0.71), indicating more reliable agreement. Distance influenced accuracy, with underestimation increasing at 75–100 cm, especially in smaller birds. Therefore, thermal imaging cannot fully replace rectal thermometry for individual-level assessment in chickens due to systematic underestimation, especially in small birds and at greater distances. However, it shows promise as a rapid, non-invasive flock-level screening tool in larger chickens when used at optimal distances (50–75 cm). The integration of thermal imaging into precision livestock farming and future farm models may enhance welfare-friendly, automated health monitoring in poultry systems.

## 1. Introduction

The accurate monitoring of body temperature is essential to evaluating health, welfare, and thermoregulation in poultry, as core body temperature is a key physiological indicator of disease, heat stress, and metabolic disturbances, making reliable measurement methods critical in both research and production settings. Additionally, body temperature regulation in chickens is influenced by several factors, including ambient temperature, humidity, age, metabolic rate, and feather insulation, with heat stress particularly impairing thermoregulation due to the lack of sweat glands and high metabolic heat production in these animals. Environmental stressors such as stocking density, ventilation, and housing systems also alter thermal balance and core temperature, underscoring the need to account for physiological and environmental variables when evaluating thermal measurement technologies [1,2]. Rectal thermometry has long been considered the conventional gold standard for measuring core body temperature in chickens. However, this method is invasive, time-consuming, and stressful for the animal, as it requires the repeated handling of birds, which can itself influence physiological responses and confound results [3,4].

Infrared thermography (IRT) offers a promising non-invasive alternative by allowing rapid and contact-free estimates of surface temperature to be obtained. It has been applied in livestock for detecting heat stress, identifying disease, and monitoring welfare status [5,6]. In poultry, IRT has been used to assess thermal responses under environmental stressors and to evaluate health conditions without direct contact [5,7]. Nevertheless, agreement between thermal and rectal temperatures is inconsistent, with discrepancies being often influenced by body size, feather coverage, surface-to-volume ratio, and environmental conditions, which can affect heat dissipation and surface temperature readings [8,9].

Technical factors also play a major role. Camera-to-object distance [10], image resolution, emissivity settings, and anatomical site selection can significantly impact accuracy and repeatability [10]. For example, measurements in larger animals often yield closer agreement between surface and core temperatures due to reduced thermal gradients, whereas in smaller animals, they show greater underestimation [11,12]. Previous work in veterinary thermography has highlighted the importance of optimizing measurement conditions to achieve reliable results, but systematic evaluations across multiple body sizes and object distances in poultry remain limited.

Beyond individual diagnostic applications, thermal imaging also aligns with the broader vision of future farm models and precision livestock farming (PLF). These concepts emphasize the real-time, automated monitoring of animal health and welfare using sensor technologies, machine vision, and data analytics. By enabling the rapid, non-invasive detection of deviations from physiological norms, thermal imaging could be integrated into PLF systems to support early disease detection, reduce handling stress, and improve overall flock management efficiency. Incorporating such technologies into commercial poultry systems is central to the development of sustainable, welfare-friendly, and data-driven farming practices [13,14].

Further work is needed to define how body size and distance affect thermal imaging accuracy in chickens. Addressing these factors is crucial to determining whether IRT can be considered interchangeable with rectal thermometry or used primarily as a flock-level screening tool.

Therefore, the present study was designed to evaluate the efficiency of thermal imaging compared with rectal thermometry for measuring chicken body temperature. Specifically, we examined how object distance (50, 75, and 100 cm) and body size (small, medium, and large) influenced agreement between thermal and rectal measurements. By systematically assessing these factors under controlled conditions, this study provides practical insights into the potential and limitations of thermal imaging as a diagnostic tool in poultry, while also highlighting its role in advancing the future farm models and precision livestock farming.

## 2. Materials and Methods

Study design

A cross-sectional, repeated-measures study was conducted to evaluate the efficiency of a thermal camera in measuring chicken body temperature, using rectal measurement as the conventional reference method for core body temperature. Each bird served as its own control and was imaged at three object distances (50, 75, and 100 cm) and classified into three body-size categories: small, S (0.30 ± 0.06 kg); medium, M (1.05 ± 0.17 kg); and large, L (2.24 ± 0.34 kg).

Animals and housing

Ninety clinically healthy Buff Sussex chickens (n = 30 per body-size group) at the production stage were enrolled from the Animal Science farm facility of King Mongkut’s Institute of Technology Ladkrabang. Birds were sex-balanced as available and housed in floor pens under standard husbandry conditions with ad libitum access to feed and water. To minimize circadian and post-prandial effects, all measurements were obtained between 08:00 and 11:00 following at least 30 min of acclimation in the imaging room, resulting in 90 observations with three repeated distances per bird (270 paired observations).

Equipment

A thermal camera (FLIR C5^®^, Astra Intersolutions Co., Ltd., Bangkok, Thailand) was used for imaging. The emissivity was set to 0.98, assuming avian comb/skin properties, with distance correction and ambient reflection compensation enabled when available. A rectal thermometer (Omron MC-246^®^, Omron Healthcare Co., Ltd., Bangkok, Thailand) was used to measure core body temperature as the reference method.

Measurement protocol

Thermal images targeted the medial canthus/eye region and comb base, which provide minimally feathered skin. For each distance, 3 consecutive images were captured during quiet stance (no preening or panting). Focus and exposure were confirmed on device; images with motion blur, open-beak panting, or obvious artifacts were discarded and repeated immediately. Video recording was performed during thermal measurements to prevent data loss. Rectal temperatures were measured before and immediately after thermal imaging at each distance (within ≤1 min) with a lubricated probe inserted 2–3 cm beyond the vent and held in contact with the mucosa until the device’s stabilization beep (~10–15 s). The average of rectal temperatures measured before and after thermal imaging was calculated and used as the individual chicken’s rectal temperature.

Data management and quality control

For each observation, we recorded bird ID, body size (S/M/L), distance (50/75/100 cm), rectal temperature (°C), and notes on behavior or artifacts. Predefined exclusion criteria were panting, wing lifting, wet plumage, visible dirt on surface measured, motion blur, or ambient drafts.

Statistical analysis

All analyses were performed in R (v. 4.5.1). Data were inspected for outliers and normality of differences, and thermal vs. rectal measurement agreement (Bland–Altman) was analyzed for each distance × size stratum, calculating bias, SD, root mean square error (RMSE), and mean absolute error (MAE). The 95% limits of agreement (LOA) were computed per stratum, and so were Lin’s concordance correlation coefficient (CCC) with bias-corrected 95% CIs and the Pearson coefficient (*r*).

Calibration modeling

A linear calibration model was developed to predict rectal temperature from thermal measurements while adjusting for body size and distance. The calibration model is specified as follows:

T_rectal_ = β_0_ + β_1_ ∗ T_thermal_ + β_2_ ∗ Size + β_3_ ∗ Distance + ϵ,

where T_rectal_ = the body temperature measured with the rectal method, T_thermal_ = the body temperature measured with the thermal camera, Size and Distance are categorical variables, and ϵ = the random error.

Accuracy Assessment

Model accuracy was assessed using coefficient estimates, *p*-values, and residual diagnostics. To evaluate screening effectiveness, calibrated IRT values were classified as normal or abnormal according to physiologically accepted temperature limits in chickens (41.0–42.0 °C). False-negative rates (FN; cases where rectal temperature was abnormal, but IRT remained normal) and the proportion of birds flagged outside the normal range were calculated for each distance–size combination.

## 3. Results

**Figure 1:**
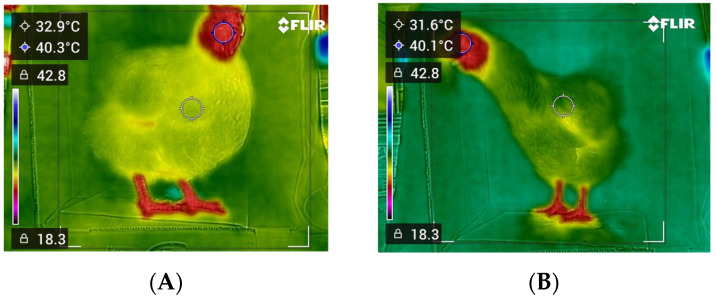
Thermographic image samples (**A**,**B**) from the present study. The blue circle and the filled blue circle with numbers indicate the body temperatures measured with the thermal camera.

Thermographic image samples obtained from the current study are shown in Figure 1, and descriptive statistics of rectal and thermal body temperatures across object distances and body sizes are presented in Table 1. Rectal temperatures were consistent across all conditions, with mean values ranging from 41.11 to 41.24 °C, small standard errors (0.06–0.07), and narrow ranges (40.43–41.95 °C), confirming the stability of the reference method. In contrast, thermal measurements varied more widely, depending on object distance and body size. At the 50 cm distance, thermal values were generally lower than rectal values in small and medium-sized birds (means of 39.88 °C and 40.45 °C, respectively), whereas in large birds, thermal readings slightly exceeded rectal values (mean of 41.27 °C), and variability was greater for thermal values, with wider ranges (e.g., 38.41–43.40 °C in large birds). At the 75 cm distance, thermal temperatures decreased further in small and medium-sized birds (means of 39.18 °C and 40.17 °C, respectively) compared with their rectal counterparts, and measurements in large birds maintained closer agreement, with a mean thermal value of 41.05 °C. At the 100 cm distance, the thermal values showed the greatest underestimation relative to rectal temperature. In small birds, the mean thermal temperature dropped to 38.59 °C, nearly 2.7 °C lower than the rectal values, and underestimation was also observed in medium-sized and large birds (means of 39.79 °C and 40.35 °C, respectively), with broader ranges indicating higher variability.

**Figure 2:**
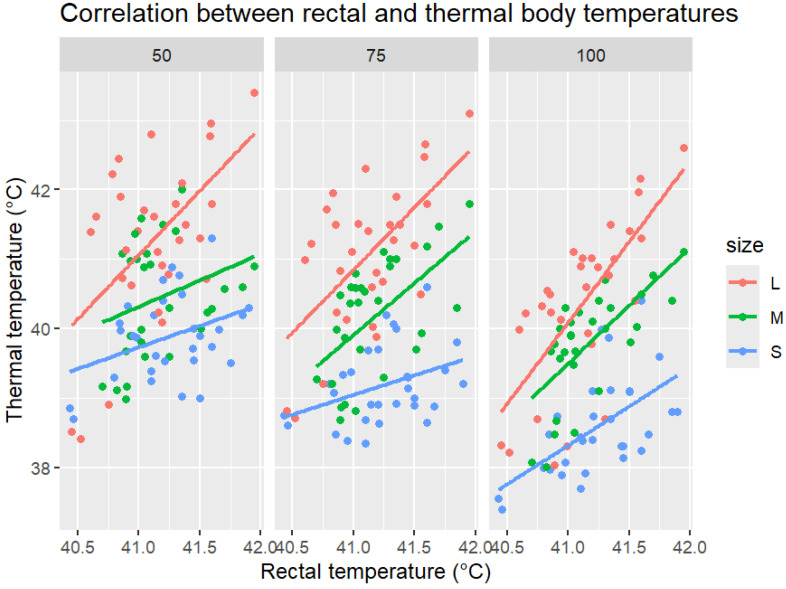
Correlation between rectal and thermal body temperatures in chickens (n = 30) measured at three object distances (50, 75, and 100 cm) and classified by body size (small = blue, medium = green, and large = red). Each point represents an individual paired measurement, with regression lines fitted separately for each body size. Measurements in large birds (red) showed the strongest correlation and the closest agreement between thermal and rectal temperatures across all distances. For medium-sized birds (green), we observed moderate correlation, while for small birds (blue), we observed consistent underestimation of rectal temperatures, which increases with greater distances.

Thermal body temperature showed a positive correlation with rectal temperature across all object distances and body sizes (Figure 2). Large-bird measurements consistently exhibited the strongest correlations, with regression slopes close to unity, indicating good agreement. For medium-sized birds, we observed moderate correlations, while for small birds, weaker associations were found, with thermal readings markedly lower than the rectal values. Increasing object distance accentuated underestimation in small and medium-sized birds, whereas for large birds, there was closer alignment with rectal temperatures, even at 100 cm.

Agreement and efficiency metrics between thermal and rectal temperatures are summarized in Table 2, where rectal temperature is used as the reference standard. At the 50 cm distance, thermal imaging consistently underestimated rectal temperature in small (bias = −1.36 °C) and medium-sized birds (−0.73 °C), with wide limits of agreement (LOAs of −2.53 to −0.19 °C and −2.29 to 0.84 °C, respectively). Errors were moderate (RMSE of 1.48–1.07 °C; MAE of 1.36–0.89 °C), and concordance correlation coefficients (CCCs) were very low (≤0.11). In large birds, the thermal values were closer to the rectal values (bias = +0.16 °C) with lower errors (RMSE of 1.04 °C; MAE of 0.84 °C), and both the correlation coefficient (*r* = 0.56, *p* < 0.01) and the CCC (0.30) improved. At the 75 cm distance, underestimation increased in small birds (bias = −2.06 °C; RMSE of 2.13 °C; MAE of 2.06 °C) with very low concordance (CCC = 0.03). In medium-sized birds, we observed smaller bias (−1.01 °C), reduced errors (RMSE of 1.23 °C), and moderate correlation (*r* = 0.56; *p* < 0.01), though the CCC remained low (0.16). In large birds, we again observed the closest agreement (bias = −0.06 °C; RMSE of 0.87 °C; MAE of 0.73 °C), with the highest correlation (r = 0.61, *p* < 0.001) and improved CCC (0.37). At the 100 cm distance, discrepancies were the most pronounced in small birds, with the largest negative bias (−2.65 °C), the highest errors (RMSE 2.72 °C, MAE 2.65 °C), and minimal concordance (CCC = 0.04), despite a moderate correlation (r = 0.57, *p* < 0.001). For medium-sized birds, we also found underestimation (bias = −1.39 °C) with moderate error (RMSE 1.51 °C) and the strongest correlation across all groups (r = 0.68, *p* < 0.001), although the CCC remained low (0.12). Large-bird measurements maintained the best performance, with relatively small bias (−0.76 °C), lower error (RMSE of 1.23 °C; MAE of 0.89 °C), high correlation (r = 0.71; *p* < 0.001), and modest concordance (CCC = 0.28).

**Figure 3:**
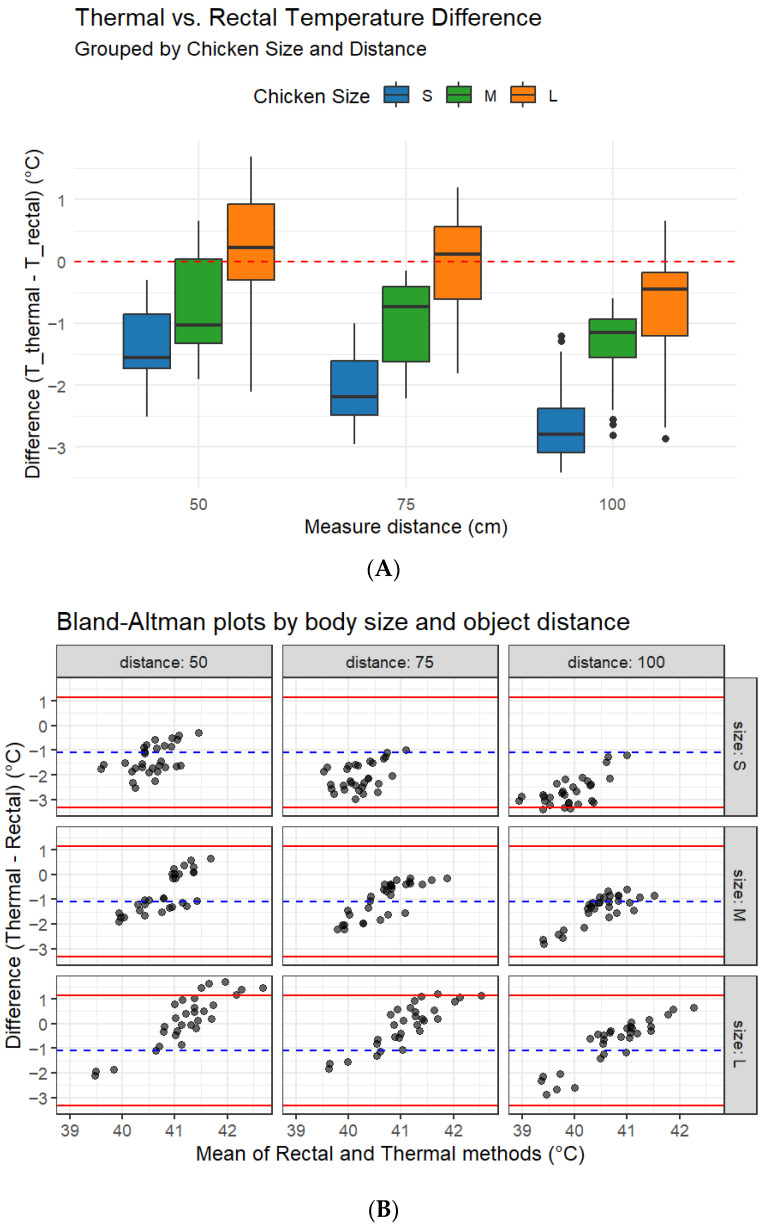
Comparison of thermal and rectal body temperature measurements in chickens (**A**) across three body sizes (S, M, and L) and three object distances (50, 75, and 100 cm). The upper panel shows box plots of temperature differences (thermal–rectal), with the red dashed line indicating zero difference (perfect agreement). Negative values represent underestimation of rectal temperature with thermal imaging. The lower panel presents Bland–Altman plots for the same conditions (**B**). The blue dashed lines represent the mean bias, and the red solid lines denote the 95% limits of agreement. In small birds, we consistently observed large negative biases; in medium-sized birds, moderate underestimation; and in large birds, minimal bias with narrower limits of agreement.

Thermal and rectal body temperature differences varied substantially with chicken size and measurement distance (Figure 3A). In small birds, thermal imaging consistently underestimated rectal temperature across all distances, with median differences ranging from −1.5 °C to −2.8 °C. The bias increased with distance, and variability was relatively narrow at 50 cm but became wider at 100 cm. In medium-sized birds, we observed smaller negative biases (−0.7 to −1.8 °C), with variability decreasing slightly as distance increased. In contrast, large-bird measurements displayed the smallest discrepancies, with differences ranging from −0.2 °C to +0.5 °C, indicating closer agreement with the rectal method. At 50 cm, in large birds, we occasionally observed a slight overestimation of rectal temperature measured with thermal imaging, while at 75 cm and 100 cm, values were close to zero bias.

Bland–Altman analysis confirmed these patterns (Figure 3B). In small birds, mean biases were consistently negative (−1.5 °C to −2.6 °C) with wide limits of agreement (up to −3.5 °C), reflecting systematic underestimation when using the thermal camera. In medium-sized birds, we observed moderate underestimation (−1.0 °C to −1.8 °C) and narrower limits of agreement compared with small birds. For large birds, the mean bias approached zero at all distances (−0.2 °C to +0.3 °C), with tighter limits of agreement, demonstrating improved reliability and consistency of thermal imaging in larger chickens. No clear proportional bias was observed with the increase in average body temperature, suggesting that differences were primarily influenced by bird size and distance rather than the absolute temperature level.

Calibration model

The linear model demonstrated a significant association between IRT and rectal temperature (*β* = 0.201; *p* < 0.001), with body size and distance also contributing to temperature prediction according to the following equation:

T_rectal_ = 32.994 + 0.211 ∗ T_thermal_ + 0.466 ∗ Size S + 0.213 * Size M − 0.192 ∗ Distance 50 − 0.112 ∗ Distance 75.

For smaller birds, we obtained higher predicted rectal temperatures than for large birds at equivalent thermal readings (size S: +0.465 °C, *p* < 0.001; size M: +0.213 °C, *p* < 0.001). Shorter measurement distances were associated with lower predicted rectal temperature (distance 50: −0.192 °C, *p* < 0.001; distance 75: −0.112 °C, *p* = 0.015) compared with 100 cm. The model explained approximately 28.9% of variation in rectal temperature (*R*^2^ = 0.289; adjusted *R*^2^ = 0.276) with a residual standard error of 0.296 °C.

Screening Performance

Screening metrics by distance and size are shown in Table 3. False-negative rates were low or zero in most conditions, indicating that calibrated IRT rarely failed to detect birds with abnormal temperatures. However, the proportion of birds classified outside the normal range was high across groups (0.567–1.00), particularly in small birds at all distances and in medium-sized birds at 75–100 cm. The most balanced performance was observed in large birds at 75 cm, where 56.7% of birds were flagged as abnormal, with a false-negative rate of 0.417. In contrast, for small birds, we observed complete over-classification at 75 and 100 cm (100% flagged as abnormal; false-negative rate = 0).

## 4. Discussion

This study evaluated the efficiency of thermal imaging compared with the conventional reference of method rectal thermometry for measuring core body temperature in chickens across different body sizes and object distances. The observed range of rectal measurements of 40.43–41.95 °C (Table 1) is consistent with the normal physiological range reported for healthy chickens (41–42 °C) [15,16,17]. Overall, the thermal measurements showed systematic underestimation compared with rectal temperature, particularly in small birds and from greater distances, where both bias and error increased substantially. In contrast, for large birds, we found the smallest biases, the lowest errors, and the strongest correlations, indicating more reliable performance of thermal imaging in this group. The results demonstrated that agreement between thermal and rectal temperatures was strongly influenced by both bird size and imaging distance. Thermal imaging consistently underestimated rectal temperature in small and medium-sized birds, particularly at greater distances, while in large birds, we observed minimal bias and higher agreement, especially at 50–75 cm. Therefore, thermal body temperature readings are distance-dependent; while very close or very far distances yield unstable or exaggerated errors, a stable plateau occurs at 40–80 cm, where attenuation remains relatively constant. This suggests that intermediate measurement distances provide the most reliable and consistent thermal readings for chickens [10].

The underestimation observed in small birds (bias of up to −2.65 °C at 100 cm) is consistent with earlier studies showing that surface temperature measurements are highly affected by body mass and insulation in poultry [18,19,20]. Smaller animals have higher surface-area-to-volume ratios, which promotes greater heat loss and increases the discrepancy between surface and core temperatures [21,22,23]. In medium-sized birds, we observed moderate biases (−0.7 to −1.8 °C), whereas large-bird thermal measurements maintained closer agreement with the rectal ones, supporting the concept that greater body mass reduces thermal gradients between surface and core tissues [24]. Object distance also played a crucial role, explained by the fact that with the increase in distance, image resolution decreases and spot size increases, leading to greater variability and underestimation in thermal readings [25]. This was particularly evident in small and medium-sized birds, whereas large-bird measurements maintained consistent agreement across 50–100 cm, suggesting that the imaging of larger surface areas is more robust to distance-related effects. These findings align with previous studies in livestock, where the accuracy of infrared thermography was the highest when the target surface area was large and the camera-to-object distance was optimized [26,27,28]. Despite moderate-to-high Pearson correlations across groups (*r* = 0.29–0.71), Lin’s concordance correlation coefficient (CCC ≤ 0.37) indicated that thermal imaging cannot yet be considered interchangeable with rectal thermometry, particularly in small birds. Similar discrepancies between correlation and concordance have been reported in veterinary thermography, underscoring the need to assess both accuracy and agreement before adopting new monitoring technologies [29,30].

This study demonstrates that infrared thermography can be calibrated to rectal temperature in chickens, with body size and distance significantly affecting predictive accuracy. The positive association between thermal and rectal temperature is consistent with previous work showing the potential of IRT as a non-invasive screening tool. However, the modest explanatory power (*R*^2^ ≈ 0.28) suggests that additional physiological or environmental factors influence thermal recordings.

A critical finding is the excellent sensitivity of calibrated IRT, reflected in near-zero false-negative rates for most distance–size combinations. This indicates strong potential for disease screening, where failure to detect febrile birds poses biosecurity risks. However, specificity was limited, as many birds were incorrectly flagged as abnormal. Over-classification was particularly evident in small birds and at greater distances, possibly due to greater heat dissipation across smaller surface areas and reduced image resolution with distance. The intermediate distance of 75 cm yielded the best balance for medium-sized and large birds, aligning with prior studies recommending moderate imaging distance to optimize accuracy and animal handling practicality. For small birds, customized calibration or close-range imaging may be required.

From a practical perspective, thermal imaging offers a rapid, non-invasive method for flock-level temperature screening. Although individual-level accuracy remains limited in small birds, the method shows promise in larger chickens and at short-to-medium distances. With further refinement, such as higher-resolution devices and improved calibration algorithms, thermal imaging could become an effective tool for precision livestock monitoring [31,32,33], reducing stress associated with invasive handling and enhancing early disease detection.

## 5. Conclusions

This study demonstrated that the accuracy of thermal imaging for estimating chicken body temperature depends strongly on bird size and imaging distance. Rectal temperature remained the most reliable reference method, with the measured values (40.43–41.95 °C) being consistent with the normal physiological range for healthy chickens. Thermal imaging consistently underestimated rectal temperature in small and medium-sized birds, particularly at greater distances, whereas in large birds, we observed minimal bias and stronger agreement with rectal measurements, especially at 50–75 cm. Although the correlation between thermal and rectal temperatures was moderate-to-high across conditions, concordance remained low, indicating that thermal imaging cannot yet replace rectal thermometry for precise individual-level assessment. Nonetheless, calibrated IRT shows promise as a rapid, non-invasive screening tool for flock-level health monitoring, particularly in larger birds and at optimal imaging distances. Its high sensitivity across measurement conditions suggests good potential for the early detection of abnormal temperature, although false-positive rates highlight the need for diagnostic confirmation with rectal measurements. Future research studies should focus on standardizing imaging distance, applying size-specific calibration models, refining image-processing algorithms, and testing performance in diseased or heat-stressed birds to further enhance accuracy and practical utility in poultry production systems.

## Figures and Tables

**Table 1:** Descriptive statistics of measured temperatures in the experimental chickens (n = 30).

Measure Distance	Body Size	Method	Mean	SE	Median	Min	Max
50	S	Rectal	41.24	0.07	41.24	40.43	41.90
		Thermal	39.88	0.11	39.90	38.70	41.30
	M	Rectal	41.17	0.06	41.06	40.70	41.95
		Thermal	40.45	0.15	40.44	38.98	42.00
	L	Rectal	41.11	0.07	41.14	40.45	41.95
		Thermal	41.27	0.22	41.40	38.41	43.40
75	S	Rectal	41.24	0.07	41.24	40.43	41.90
		Thermal	39.18	0.10	39.11	38.34	40.60
	M	Rectal	41.17	0.06	41.06	40.70	41.95
		Thermal	40.17	0.15	40.37	38.68	41.80
	L	Rectal	41.11	0.07	41.14	40.45	41.95
		Thermal	41.05	0.19	41.21	38.71	43.10
100	S	Rectal	41.24	0.07	41.24	40.43	41.90
		Thermal	38.59	0.13	38.46	37.40	40.40
	M	Rectal	41.17	0.06	41.06	40.70	41.95
		Thermal	39.79	0.14	39.96	38.01	41.10
	L	Rectal	41.11	0.07	41.14	40.45	41.95
		Thermal	40.35	0.22	40.53	38.03	42.60

**Table 2:** Efficiency comparison between thermal and rectal temperature measurements in experimental chickens (n = 30).

Measure Distance	Body Size	Bias (°C)	95% LOA (°C)	RMSE (°C)	MAE (°C)	Pearson r (95% CI)	CCC (95% CI)
50	S	−1.36	−2.53 to −0.19	1.48	1.36	0.36 (0.00–0.64) *	0.07 (−0.00–0.14)
	M	−0.73	−2.29 to 0.84	1.07	0.89	0.29 (−0.08–0.59)	0.11 (−0.03–0.25)
	L	0.16	−1.89 to 2.20	1.04	0.84	0.56 (0.24–0.76) **	0.30 (0.13–0.45)
75	S	−2.06	−3.14 to −0.99	2.13	2.06	0.37 (0.01–0.64) *	0.03 (−0.00–0.06)
	M	−1.01	−2.42 to 0.40	1.23	1.01	0.56 (0.25–0.76) **	0.16 (0.05–0.27)
	L	−0.06	−1.80 to 1.67	0.87	0.73	0.61 (0.33–0.80) ***	0.37 (0.20–0.53)
100	S	−2.65	−3.81 to −1.50	2.72	2.65	0.57 (0.27–0.77) ***	0.04 (0.01–0.07)
	M	−1.39	−2.59 to −0.19	1.51	1.39	0.68 (0.42–0.83) ***	0.12 (0.05–0.20)
	L	−0.76	−2.67 to 1.15	1.23	0.89	0.71 (0.46–0.85) ***	0.28 (0.15–0.41)

Bias = mean (thermal–rectal). LOA = limit of agreement (bias ± 1.96 × SD). *, **, and *** statistical significance at *p* < 0.5, *p* < 0.01, and *p* < 0.001, respectively.

**Table 3:** Screening accuracy for calibrated infrared thermography by measurement distance and chicken size (n = 30).

Distance (cm)	Size	False-Negative Rate	Proportion Flagged Outside Normal Range
100	Large	0.000	0.733
100	Medium	0.000	0.967
100	Small	0.000	1.000
75	Large	0.417	0.567
75	Medium	0.000	0.800
75	Small	0.000	1.000
50	Large	0.417	0.567
50	Medium	0.300	0.700
50	Small	0.000	0.967

Notes: Normal temperature threshold = 41.0–42.0 °C. False-negative rate = proportion of birds with abnormal rectal temperature misclassified as normal with calibrated IRT.

## Data Availability

The original contributions presented in the study are included in the article. Further inquiries can be directed to the corresponding author.

## Abbreviations

The following abbreviations are used in this manuscript:

CCC	Concordance correlation coefficient
CI	Confidence interval
IRT	Infrared thermography
LOA	Limit of agreement
MEA	Mean absolute error
RMEA	Root mean square error
SD	Standard deviation
